# Driving pressure-guided ventilation decreases the mechanical power compared to predicted body weight-guided ventilation in the Acute Respiratory Distress Syndrome

**DOI:** 10.1186/s13054-022-04054-5

**Published:** 2022-06-20

**Authors:** Anne-Fleur Haudebourg, Samuel Tuffet, François Perier, Keyvan Razazi, Nicolas de Prost, Armand Mekontso Dessap, Guillaume Carteaux

**Affiliations:** 1 CHU Henri Mondor-Albert Chenevier, Service de Médecine Intensive Réanimation, Assistance Publique- Hôpitaux de Paris, 51, Avenue du Maréchal de Lattre de Tassigny, 94010 Créteil Cedex, France; 2 Groupe de Recherche Clinique CARMAS, Faculté de Santé, Université Paris Est-Créteil, 94010 Créteil Cedex, France; 3 INSERM U955, Institut Mondor de Recherche Biomédicale, 94010 Créteil Cedex, France

**Keywords:** Acute respiratory distress syndrome, Mechanical ventilation, Protective ventilation, Tidal volume, Driving pressure, Ventilator-induced lung injury, Mechanical power

## Abstract

**Background:**

Whether targeting the driving pressure (∆P) when adjusting the tidal volume in mechanically ventilated patients with the acute respiratory distress syndrome (ARDS) may decrease the risk of ventilator-induced lung injury remains a matter of research. In this study, we assessed the effect of a ∆P-guided ventilation on the mechanical power.

**Methods:**

We prospectively included adult patients with moderate-to-severe ARDS. Positive end expiratory pressure was set by the attending physician and kept constant during the study. Tidal volume was first adjusted to target 6 ml/kg of predicted body weight (PBW-guided ventilation) and subsequently modified within a range from 4 to 10 ml/kg PBW to target a ∆P between 12 and 14 cm H_2_O. The respiratory rate was then re-adjusted within a range from 12 to 40 breaths/min until EtCO_2_ returned to its baseline value (∆P-guided ventilation). Mechanical power was computed at each step.

**Results:**

Fifty-one patients were included between December 2019 and May 2021. ∆P-guided ventilation was feasible in all but one patient. The ∆P during PBW-guided ventilation was already within the target range of ∆P-guided ventilation in five (10%) patients, above in nine (18%) and below in 36 (72%). The change from PBW- to ∆P-guided ventilation was thus accompanied by an overall increase in tidal volume from 6.1 mL/kg PBW [5.9–6.2] to 7.7 ml/kg PBW [6.2–8.7], while respiratory rate was decreased from 29 breaths/min [26–32] to 21 breaths/min [16–28] (*p* < 0.001 for all comparisons). ∆P-guided ventilation was accompanied by a significant decrease in mechanical power from 31.5 J/min [28–35.7] to 28.8 J/min [24.6–32.6] (*p* < 0.001), representing a relative decrease of 7% [0–16]. With ∆P-guided ventilation, the PaO_2_/FiO_2_ ratio increased and the ventilatory ratio decreased.

**Conclusion:**

As compared to a conventional PBW-guided ventilation, a ∆P-guided ventilation strategy targeting a ∆P between 12 and 14 cm H_2_O required to change the tidal volume in 90% of the patients. Such ∆P-guided ventilation significantly reduced the mechanical power. Whether this physiological observation could be associated with clinical benefit should be assessed in clinical trials.

## Background

The major goal of lung-protective ventilation in the Acute Respiratory Distress Syndrome (ARDS) is to reduce ventilator/ventilation-induced lung injuries (VILI) by minimizing strain and stress applied to the lung by mechanical ventilation [1, 2]. As ARDS patients are characterized by a dramatical decrease in aerated lung volume, lowering the tidal volume (*V*_T_) down to 6 ml/kg of predicted body weight (PBW) has been shown more than 20 years ago to improve their survival [3] and has become the cornerstone of lung protective ventilation [4]. In such patients, the decrease in respiratory system compliance (*C*_RS_) is correlated to the decrease in the lung volume available for ventilation [5–7]. The driving pressure (ΔP), defined as the difference between the plateau pressure and the positive end expiratory pressure (PEEP), represents the ratio between the *V*_T_ and the *C*_RS_ [8]. Therefore, the ΔP normalizes the *V*_T_ to a surrogate of the aerated lung, rather than to the theoretical lung size as is the case when *V*_T_ is related to PBW. In a post hoc analysis of nine randomized control trials, Amato et al*.* found that high ΔP was a better predictor of mortality than high *V*_T_ [9], with an increased risk of death when the ΔP exceeded 14 cm H_2_O. These findings have been confirmed in a subsequent meta-analysis [10] and reinforced by large-scale observational data [11–13]. More recently, it has been shown that the mortality benefit of lowering *V*_T_ in ARDS varied according to the *C*_RS_: the patients with lower *C*_RS_ were likely to have a greater mortality benefit, whereas patients with higher *C*_RS_ were likely to have a lower mortality benefit [14]. All these data argue for targeting ΔP rather than *V*_T_ scaled to PBW in ARDS.

In this study, we hypothesized that a ΔP-guided tidal ventilation strategy (ΔP-Vent), targeting a ΔP between 12 and 14 cm H_2_O, may reduce the risk of VILI as compared to a PBW-guided tidal ventilation strategy (PBW-Vent) in ARDS patients. Such ∆P-vent approach could protect against excessive driving pressure in patients with lower *C*_RS_ while permitting higher driving pressure in patients with higher *C*_RS_, allowing a concomitant decrease in the respiratory rate. Interestingly, recent data suggest that both driving pressure and respiratory rate are predictors of mortality in ARDS [15]. Their combined effect may depend on *C*_RS_ with potential value in increasing tidal volume while decreasing respiratory rate in patients with higher *C*_RS_. The mechanical power represents the energy applied to the respiratory system by the ventilator and has been considered as a surrogate for the risk of VILI [16–18]. It is associated with mortality during controlled mechanical ventilation in ARDS [15, 19] and has the advantage of taking into account both an elastic component related to the driving pressure and the possible impact of respiratory rate. Thus, the aim of our study was to compare, in ARDS patients, the mechanical power resulting from a ΔP-Vent versus that resulting from a PBW-Vent.

## Methods

### Study design

This is an ancillary report of an ongoing prospective monocentric observational study on ventilator settings and respiratory mechanics assessment in patients with ARDS, conducted in the Henri Mondor University Hospital medical ICU, Créteil, France. The study was approved by the ethics committee “CPP Sud Ouest et Outre Mer III” (2018-A00867–48). According to the French law, non-opposition to participate in the study from the patient or his/her next of kin was collected prior to inclusion.

### Patients

Inclusion criteria were as follows: moderate-to-severe ARDS according to the Berlin definition [20], less than 5 days since the onset of ARDS, and passive ventilation, i.e., no clinical detection of spontaneous respiratory effort. Non-inclusion criteria were the followings: age < 18 years, pregnancy, extra-corporeal membrane oxygenation, the need for strict control of PaCO_2_ (e.g., severe neurological impairment) and patients under law protection. Exclusion criteria was the impossibility to achieve ΔP-Vent (see below).

### Ventilator settings

Positive end-expiratory pressure (PEEP) and fraction of inspired oxygen (FiO_2_) were set by the attending physician and kept constant during the study. As per our clinical routine (see below), the PEEP set by the clinician was equal or above the airway opening pressure, while ensuring a plateau pressure below 30 cm H_2_O. The tidal volume was routinely set at 6 mL/kg of PBW, with the respiratory rate adjusted to maintain the pH between 7.20 and 7.45 [21, 22], which corresponds to the PBW-Vent. Respiratory mechanics was assessed during PBW-Vent, including the ΔP (see below). The principle of the ΔP-Vent was to target a ΔP between 12 and 14 cm H_2_O. Thus, if the ΔP measured during PBW-Vent was below 12 or above 14 cm H_2_O, the tidal volume was readjusted within a range from 4 to 10 ml/kg PBW to target a ΔP between 12 and 14 cm H_2_O without exceeding a plateau pressure of 30 cm H_2_O. The respiratory rate was then modified within a range from 12 to 40 breaths/min until the end-tidal CO_2_ (EtCO_2_) returned to its baseline value observed during PBW-Vent, defining the ΔP-Vent.

### Measurements

At baseline during PBW-Vent, the potential airway closure phenomenon was detected by measuring the airway opening pressure during a low flow (< 6 L/min) insufflation, as previously described [23]. The potential for lung recruitment was assessed using recruitment-to-inflation ratio (R/I ratio) obtained with a drop in PEEP over a single breath maneuver, as previously detailed [23]. By default, R/I ratio was assessed between 15 and 5 cmH_2_O of PEEP. However, in case of airway closure, the low PEEP was set above the airway opening pressure. Ventilator’s settings and respiratory mechanics were collected during PBW-Vent and after at least 30 min of ΔP-Vent application. For that purpose, a 0.3 s end-inspiratory pause and a one to two second end-expiratory occlusion maneuver were performed to record both the plateau pressure and total PEEP. From these data, the mechanical power (in Joules per minute) was computed as the algebraic sum of elastic and resistive power [15, 16]:

$$\begin{aligned} & {\text{Elastic power}}\,({\text{J}}/\min ): \, \,0.098 \, \times {\text{ RR }} \times \, V_{T} \times \, \left( {{\text{PEEP }} + \, 0.5 \, \times \, \Delta P} \right) \\ & {\text{Resistive power}}\,({\text{J}}/\min ):\, \, 0.098 \, \times {\text{ RR }} \times \, V_{T} \times \, \left( {{\text{Ppeak }}{-}{\text{ Pplat}}} \right) \\ \end{aligned}$$

where RR is the respiratory rate, *V*_T_ the tidal volume, Ppeak the peak airway pressure, and Pplat the plateau pressure.

Blood gases were performed under PBW-Vent and after 30 min of ΔP-Vent if an arterial line was present.

A transthoracic echocardiography was conducted under PBW-Vent and ΔP-Vent for the last 24 patients included in the study in order to assess the potential effects of ΔP-Vent on cardiac output and right ventricle function. These echocardiography tests were performed using an S7 or E9 ultrasound system (GEMS, Buc, France). Pulsed-wave Doppler were obtained at aortic valve to assess aortic velocity–time integral for cardiac output computation. Right ventricle preload was assessed using the maximal diameter of inferior vena cava on long-axis M-mode view. Right ventricle systolic function assessment relied on tricuspid annulus plane systolic excursion (TAPSE) and on tissue Doppler peak systolic wave at tricuspid lateral annulus (tricuspid S’ wave). Relative size of right and left ventricles was assessed on apical four-chamber view using the end-diastolic right ventricle/left ventricle area ratio. The systolic pulmonary arterial pressure was assessed using the peak velocity of tricuspid regurgitation on continuous-wave Doppler.

### Endpoints

The primary endpoint was the mechanical power. Secondary endpoints were the followings: mechanical power normalized to PBW (norMP), ΔP, tidal volume, respiratory rate, minute ventilation, compliance and resistance of the respiratory system, PaO_2_/FiO_2_ ratio, PaCO_2_, ventilatory ratio [24], cardiac output and right ventricle function.

### Statistics

Data were analyzed using SPSS Base 20.0 statistical software package (SPSS, Chicago, IL). Sample size calculation was made using the “pwr” package in R (version 4.0.1). To be able to show a potential difference in mechanical power between *V*_T_-Vent and ΔP-Vent with a medium effect size—the effect size being the difference between the means divided by the pooled standard deviation; a value of 0.5 defining a medium effect size for paired comparisons [25]—a type I error of 0.05 and a statistical power of 90%, 44 patients were needed using two-sided tests. We therefore chose to include at least 50 patients. Continuous data were expressed as medians (25th–75th percentiles) and compared using Mann–Whitney test for independent variables and Wilcoxon signed rank test for related variables. Categorical variables, expressed as percentages, were evaluated using Chi-square or Fisher exact tests as appropriate. A *p* < 0.05 was considered significant.

## Results

### Patients’ characteristics

Fifty-one patients were included between December 2019 and May 2021. One patient was secondarily excluded because ΔP-Vent was impossible to achieve with the predefined boundaries of *V*_T_ (≥ 4 mL/kg) and RR (≤ 40/min). ΔP-Vent was feasible in the remaining 50 (98%) patients, whose main characteristics are reported in Table 1. The basal ΔP during PBW-Vent was already within the target range of ΔP-Vent in five patients (10%), above in nine (18%) and below in 36 (72%). Thus, the *V*_T_ was left unchanged in five patients, decreased in nine and increased in 36. In all patients in whom tidal volume was increased, the increase was possible to meet the target ΔP before the plateau pressure limit was reached.

**Table 1:** Characteristics of patients who underwent predicted body weight- and driving pressure-guided ventilation

	All patients (*n* = 50)
Age, years	63 [55–70]
Sex ratio, M/W	33/17
BMI, kg/m^2^	30 [25–32]
Reasons for intubation, *n* (%)	
de novo AHRF	44 (88%)
Covid-19 related AHRF	37 (74%)
COPD exacerbation	2 (4%)
Shock	7 (14%)
Cardiac arrest	4 (8%)
Coma	3 (6%)
SAPS II at admission	40 [31–56]
SOFA at admission	6 [3–8]
SOFA at inclusion	7 [3–8]
MV duration before inclusion, days	1 [1–3]
ARDS duration before inclusion, days	1 [1–3]

*BMI* Body Mass Index, *MV* mechanical ventilation, *AHRF* acute hypoxemic respiratory failure, *COPD* chronic obstructive pulmonary disease, *SAPS* Simplified Acute Physiology Score [26], *SOFA* Sepsis-related Organ Failure Assessment [27], *ARDS* Acute Respiratory Distress Syndrome

### Primary endpoint

**Fig. 1:**
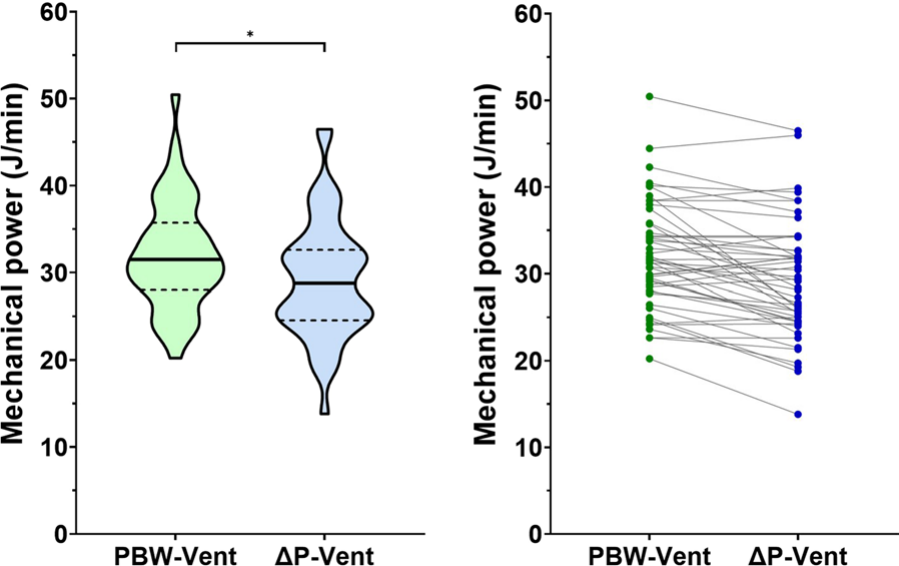
Change in mechanical power between predicted body weight-guided ventilation (PBW-Vent) and driving pressure-guided ventilation (ΔP-Vent). **A** The violin plots represent the mechanical power (thick horizontal line: median; thin horizontal dashed lines: 25th and 75th percentiles). *Denotes statistical significance. **B** Individual data

Compared to PBW-Vent, ΔP-Vent was accompanied by a significant decrease in mechanical power from 31.5 J/min [28–35.7] to 28.8 J/min [24.6–32.6] (*p* < 0.001, Table 2 and Fig. 1), corresponding to a relative decrease of 7% [0–16].

**Table 2:** Ventilatory and hemodynamic changes between tidal volume-guided ventilation and driving pressure-guided ventilation

	PBW-guided ventilation	ΔP-guided ventilation	*p*
FiO_2_, %	50 [40–60]	50 [40–60]	1.00
PEEP, cm H_2_O	10 [8–12]	10 [8–12]	1.00
Tidal volume, mL/kg PBW	6.1 [5.9–6.2]	7.7 [6.2–8.7]	< 0.001
Respiratory rate, breaths/min	29 [26–32]	21 [16–28]	< 0.001
Minute ventilation, L/min	11.3 [9.7–12.7]	10.2 [8.9–11.6]	< 0.001
EtCO_2_, mm Hg	30 [27–36]	30 [27–37]	0.16
Plateau pressure, cm H_2_O	22 [19–25]	24 [22–26]	< 0.001
Auto-PEEP, cm H_2_O	1 [1–1.25]	1 [0–1]	0.002
Driving pressure, cm H_2_O	10 [9–13]	13 [12–14]	< 0.001
Airways resistance, cm H_2_O/L/s	13 [9–16]	13 [9–16]	0.71
Respiratory system compliance, mL/cm H_2_O	38 [27–48]	40 [27–48]	0.15
Mechanical power, J/min	31.5 [28–35.7]	28.8 [24.6–32.6]	< 0.001
Elastic power, J/min	18.4 [15.1–20.9]	16.6 [13.7–20.9]	< 0.001
Resistive power, J/min	14.1 [9.9–16.6]	12.0 [9.7–13.8]	< 0.001
NorMP, J/min/kg	0.50 [0.43–0.58]	0.46 [0.36–0.55]	< 0.001
SpO_2_, %	96 [93–97]	95 [94–97]	0.72
PaO_2_/FiO_2_	126 [107–157]	135 [104–172]	0.005
PaCO_2_, cm H_2_O	42 [37–48]	42 [35–48]	0.07
Alveolar dead space, %	26 [16–34]	29 [15–34]	0.07
Ventilatory ratio	1.97 [1.66–2.46]	1.77 [1.40–2.27]	< 0.001
Heart rate, beats/min	71 [64–85]	72 [63–88]	0.34
Mean arterial pressure, mm Hg	80 [72–88]	81 [71–88]	0.61
Lactate, mmol/L	1.4 [1–2.2]	1.5 [1–2.1]	0.70
*Transthoracic echocardiography (n* = *24)*			
Cardiac output, L/min	4.2 [3.6–4.8]	4.1 [3.8–5.1]	0.97
RV surface, cm^2^	15 [13–17]	15 [13–17]	0.76
RV/LV ratio	0.60 [0.50–0.61]	0.60 [0.51–0.68]	0.74
Tricuspid S’ wave, cm/s	15.5 [11.3–17.5]	14.2 [11.1–17]	0.02
TAPSE, mm	19 [17–22]	20 [17–23]	0.10
PAPs, mm Hg	38 [31–46]	38 [29–42]	0.28
Inferior vena cava, mm	22 [16–24]	21 [18–23]	0.73

*PBW* predicted body weight, *ΔP* driving pressure, *FiO*_*2*_ inspired fraction of oxygen, *PEEP* positive end expiratory pressure, *EtCO2* end-tidal CO2, *NorMP* mechanical power normalized to predicted body weight, *RV* right ventricle, *LV* left ventricle. *TAPSE* tricuspid annular plane systolic excursion, *PAPs* systolic pulmonary arterial pressure

Individual data showed that the mechanical power strictly decreased with ΔP-Vent in 36 patients (from 31.5 J/min [27.8–35.8] to 26.4 J/min [24.1–32.9]), and remained unchanged or increased in 14 patients (from 30.7 J/min [28.7–35.4] to 31.9 J/min [30.4–35.4]).

### Secondary endpoints

Switching from PBW-Vent to ΔP-Vent required a significant increase in *V*_T_, and a significant decrease in respiratory rate (Table 2). As compared to PBW-Vent, ΔP-Vent was characterized by a significant increase in ΔP, plateau pressure and PaO_2_/FiO_2_ ratio, and a significant decrease in minute ventilation, auto-PEEP, and ventilatory ratio, while PaCO_2_ was similar between the two ventilation strategies (Table 2). The hemodynamic parameters, cardiac output and right ventricle function were not altered by the change in ventilation strategy.

**Fig. 2:**
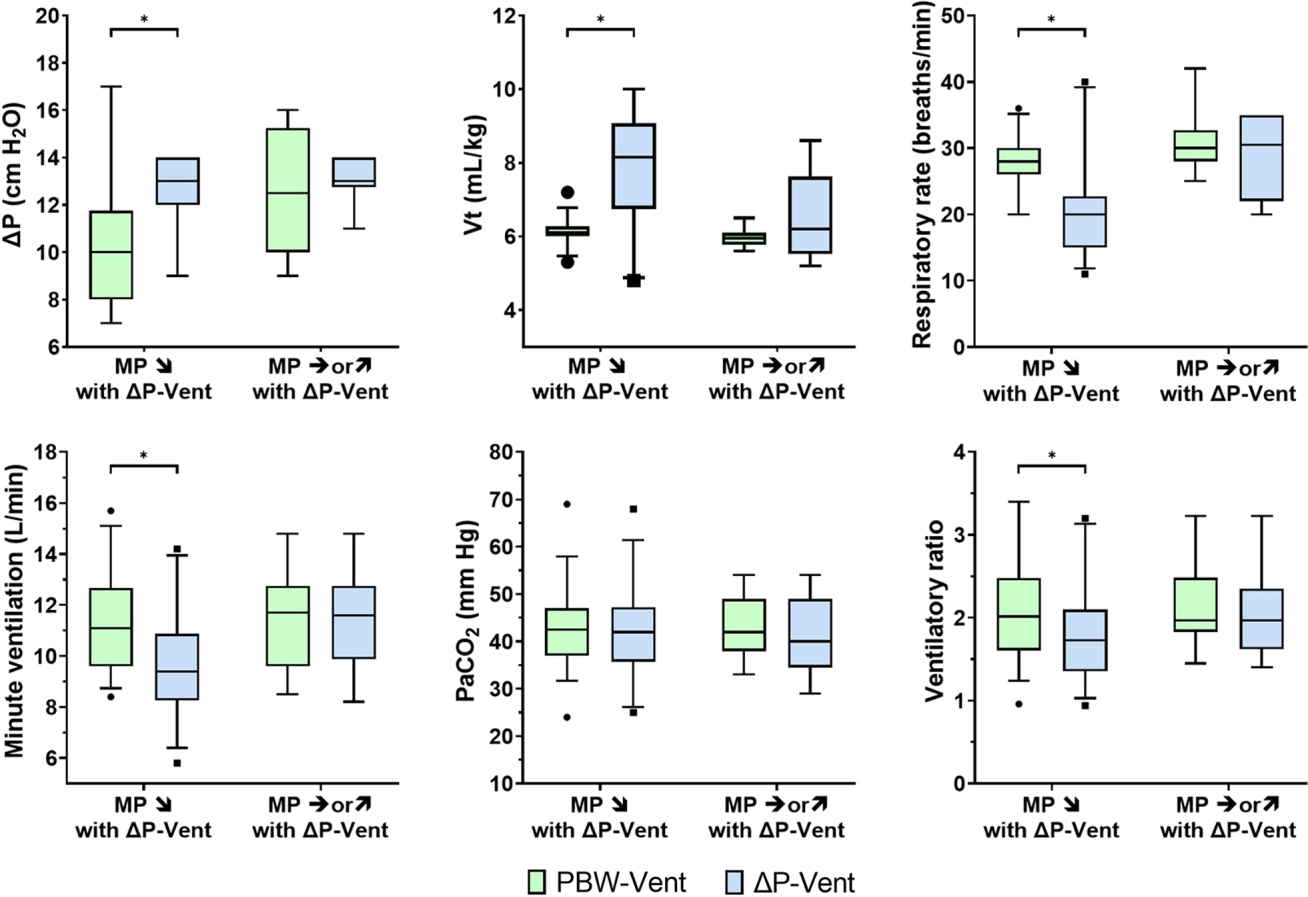
Change in ventilatory parameters according to the change in mechanical power with ΔP-Vent. MP ↘ with ΔP-Vent: patients in whom the mechanical power strictly decreased with ΔP-Vent compared with PBW-Vent (*n* = 36). MP → or ↗ with ΔP-Vent: patients in whom the mechanical power remained unchanged or increased with ΔP-Vent compared with PBW-Vent (*n* = 14). ΔP: driving pressure; Vt: tidal volume. *Denotes statistical significance

At baseline during PBW-Vent, patients whose mechanical power decreased with ΔP-Vent (*n* = 36) had a significantly higher SAPS II, SOFA score and respiratory system compliance and a significantly lower driving pressure, plateau pressure, R/I ratio and alveolar dead space (Table 3). In these patients, switching to ΔP-Vent was accompanied by a significant increase in ΔP and *V*_T_ and a significant decrease in respiratory rate, minute ventilation and ventilatory ratio (Fig. 2). In patients whose mechanical power did not decrease with ΔP-Vent (*n* = 14), applying ΔP-Vent was not associated with a significant change in respiratory pattern or ventilatory ratio compared with PBW-Vent (Fig. 2).

**Table 3:** Baseline and ventilatory characteristics according to the evolution of mechanical power with driving pressure-guided ventilation

	MP strictly decreased with ∆P-Vent (*n* = 36)	MP increased with ∆P-Vent (*n* = 14)	*p*
Age, years	64 [55–70]	63 [52–69]	0.97
BMI, kg/m^2^	30 [24–32]	28 [25–32]	0.70
SAPS II at admission	43 [35–61]	31 [26–39]	0.002
SOFA at admission	7 [4–10]	3 [2–5]	< 0.001
SOFA at inclusion	7 [3–10]	6 [3–8]	0.41
FiO_2_, %	60 [50–78]	70 [50–85]	0.30
PEEP, cm H_2_O	9 [8–12]	11 [8–12]	0.40
Tidal volume, mL/kg PBW	6.1 [6.0–6.2]	6.0 [5.8–6.1]	0.11
Respiratory rate, breaths/min	28 [26–30]	30 [28–33]	0.19
Minute ventilation, L/min	11.1 [9.6–12.7]	11.7 [9.6–12.8]	1.0
EtCO_2_, mm Hg	33 [27–40]	29 [24–32]	0.07
Plateau pressure, cm H_2_O	20 [18–24]	24 [22–26]	0.03
Driving pressure, cm H_2_O	10 [8–12]	13 [10–15]	0.02
Airways resistance, cm H_2_O/L/s	13 [11–16]	10 [8–18]	0.27
Respiratory system compliance, mL/cmH_2_O	40 [32–50]	27 [23–43]	0.02
R/I ratio	0.25 [0.1–0.42]	0.43 [0.26–0.5]	0.02
Mechanical power, J/min	31.5 [27.8–35.8]	30.8 [28.7–35.4]	0.92
NorMP, J/min/kg	0.50 [0.43–0.57]	0.51 [0.45–0.62]	0.39
SaO_2_, %	95 [93–97]	94 [92–98]	0.96
PaO_2_/FiO_2_, mm Hg	127 [109–162]	121 [82–154]	0.17
PaCO_2_, cmH_2_O	43 [37–47]	42 [38–49]	0.85
Alveolar dead space, %	22 [15–31]	33 [22–44]	0.03
Ventilatory ratio	2.02 [1.6–2.48]	1.97 [1.83–2.48]	0.59

*MP* mechanical power, *ΔP-Vent* driving pressure-guided ventilation, *PBW* predicted body weight, *FiO*_*2*_ inspired fraction of oxygen, *PEEP* positive end expiratory pressure, *EtCO2* end-tidal CO_2_, *NorMP* mechanical power normalized to predicted body weight

## Discussion

The main findings of our study are the followings: a ∆P-guided ventilation targeting a ∆P between 12 and 14 cm H_2_O represented a distinct strategy from a conventional PBW-guided ventilation as it required *V*_T_ changes in 90% of the patients; the direction of the change in *V*_T_ was an increase in the majority of cases (72%), accompanied by a concomitant decrease in respiratory rate; such ∆P-guided ventilation led to a significant decrease in mechanical power while PaO_2_/FiO_2_ and ventilatory ratio improved; the decrease in mechanical power was mainly driven by patients with higher respiratory system compliance, thus in whom the *V*_T_ increased during ∆P-guided ventilation.

A ΔP-limited strategy (aiming at minimizing the ΔP) has been proposed and seems feasible [28]. However, decreasing the ΔP at the price of an increase in respiratory rate may be at higher risk of unfavorable outcome in patients with higher compliance [19]. In this study, we rather assessed a ΔP-Vent strategy targeting a ΔP range below the threshold identified as associated with an increased risk of death. If the ΔP was above the threshold during PBW-Vent, the *V*_T_ was decreased in order to avoid excessive strain. However, the ΔP at baseline during PBW-Vent in our series was below the predefined target range in a majority of patients, in accordance with values usually observed in ARDS patients when the *V*_T_ is set at 6–8 ml/kg of PBW [11]. *V*_T_ was therefore most often increased to achieve ΔP-Vent. One may assume that allowing some increase in *V*_T_ in patients with higher compliance during the ΔP-Vent strategy could be associated with favorable physiological effects as promotion of recruitment and decrease in alveolar dead space [29], improvement of oxygenation [30], unloading of respiratory muscles, attenuation of respiratory drive [31, 32], relief of dyspnea [14], decreased risk of occurrence of breath stacking [33, 34] and decreased need for sedative drugs [35]. Of note, in our study, ΔP-Vent was accompanied by an increase in PaO_2_/FiO_2_ ratio, and a decrease in ventilatory ratio. Whether such physiological effects could be associated with improvement in clinical outcome warrants further research.

In our study, ΔP-Vent was associated with a significant decrease in both the resistive and elastic component of the mechanical power, as compared to PBW-Vent. Mechanical power, which represents the energy delivered to the respiratory system, could be considered as a target for VILI prevention [18]. Cressoni et al*.* conducted an experimental study on piglets suggesting that neither the *V*_T_ alone nor the respiratory rate could generate VILI, which instead was induced by their combination when the mechanical power produced was higher than a certain threshold [17]. Therefore, paying attention to mechanical power might help broaden our focus on VILI, taking into account not only *V*_T_ and ΔP, but also respiratory rate and their combination. In an analysis of more than 8000 critically ill patients from the MIMIC-III and eICU databases, Serpa Neto et al*.* retrieved that high mechanical power was independently associated with high in-hospital mortality, even at low tidal volumes [19]. More recently, a retrospective analysis of 4549 patients included in six randomized clinical trials of protective ventilation showed that mechanical power was a significant predictor of mortality at 28 or 60 days [15]. In our study, the ΔP-Vent induced a relative decrease in mechanical power of 7% [0–16]. In a prospective cohort study involving 13,408 patients, Urner et al*.* reported a significant increase in the hazard of death with each daily increment in mechanical power over the whole duration of mechanical ventilation, suggesting that even a small decrease in mechanical power could be relevant if maintained over time. However, a causal relationship between mechanical power and clinical outcome has not been demonstrated to date. High mechanical power may be a marker of lung injury rather than inappropriate ventilator settings. Indeed, during conventional protective ventilation, the mechanical power increases in case of *C*_RS_ impairment and decreases during resolution of the lung disease. Thus, the clinical impact of a ventilation strategy that is accompanied by a decrease in mechanical power remains unclear and deserves future clinical trials.

Our study has some limitations. First, this was a single-center study with a significant proportion of COVID-19-related ARDS among the included patients. Conflicting data have been reported about potential differences in the respiratory system compliance in the early phase of COVID-19-related ARDS compared to ARDS of other origins [36–40]. However, the ΔP at baseline during PBW-Vent in our population was consistent with reports prior to COVID-19 pandemics [11], suggesting external validity. Second, PEEP management in our unit may have influenced the value of mechanical power. In fact, mechanical power is modeled with a positive linear relationship with PEEP. However, we did not modify PEEP between PBW-Vent and ΔP-Vent. The difference in mechanical power was therefore exclusively related to variations in *V*_T_ and respiratory rate, and should be reproducible as long as the PEEP level remains of the same order of magnitude. Third, our PEEP management may also have influenced the plateau pressure values. Excessive plateau pressure may be associated with higher mortality even with limited driving pressure [41]. In our study however, the plateau pressure remained below 30 cm H_2_O during both ventilation strategies in all patients. Lastly, the ΔP value is influenced by the chest wall compliance and a high ΔP may be related to a low chest wall compliance rather than an excessive lung strain. We did not record the esophageal pressure and were thus unable to measure the chest wall compliance. However, we aimed at assessing a pragmatic approach that could be easily translated into clinical practice. As the ΔP-Vent was feasible and different from PBW-Vent in 90% of our patients, designing clinical trials comparing the two strategies is attainable.

## Conclusion

As compared to a conventional PBW-guided ventilation, a ∆P-guided ventilation strategy targeting a ∆P between 12 and 14 cm H_2_O significantly reduced the mechanical power.

## Data Availability

The datasets used and/or analyzed during the current study are available from the corresponding author on reasonable request.
